# Proteomic Analysis and Virulence Assessment of *Granulicatella adiacens* Secretome

**DOI:** 10.3389/fcimb.2019.00104

**Published:** 2019-04-24

**Authors:** Maribasappa Karched, Radhika G. Bhardwaj, Ali Tiss, Sirkka Asikainen

**Affiliations:** ^1^Oral Microbiology Research Laboratory, Faculty of Dentistry, Kuwait University, Kuwait City, Kuwait; ^2^Functional Proteomics and Metabolomics Unit, Dasman Diabetes Institute, Kuwait City, Kuwait

**Keywords:** *Granulicatella*, secretome, oral, infective endocarditis, virulence, periodontitis

## Abstract

Despite reports on the occurrence of *Granulicatella adiacens* in infective endocarditis, few mechanistic studies on its virulence characteristics or pathogenicity are available. Proteins secreted by this species may act as determinants of host-microbe interaction and play a role in virulence. Our aim in this study was to investigate and functionally characterize the secretome of *G. adiacens*. Proteins in the secretome preparation were digested by trypsin and applied to nanoLC-ESI-MS/MS. By using a combined mass spectrometry and bioinformatics approach, we identified 101 proteins. Bioinformatics tools predicting subcellular localization revealed that 18 of the secreted proteins possessed signal sequence. More than 20% of the secretome proteins were putative virulence proteins including serine protease, superoxide dismutase, aminopeptidase, molecular chaperone DnaK, and thioredoxin. Ribosomal proteins, molecular chaperones, and glycolytic enzymes, together known as “moonlighting proteins,” comprised fifth of the secretome proteins. By Gene Ontology analysis, more than 60 proteins of the secretome were grouped in biological processes or molecular functions. KEGG pathway analysis disclosed that the secretome consisted of enzymes involved in biosynthesis of antibiotics. Cytokine profiling revealed that secreted proteins stimulated key cytokines, such as IL-1β, MCP-1, TNF-α, and RANTES from human PBMCs. In summary, the results from the current investigation of the *G. adiacens* secretome provide a basis for understanding possible pathogenic mechanisms of *G. adiacens*.

## Introduction

*Granulicatella adiacens* is part of the normal microbiota in the oral cavity, urogenital, and intestinal tract (Ruoff, 1991). It can occasionally cause serious infections such as infective endocarditis (Lin and Hsu, 2007), but may also participate in periodontitis (Belstrøm et al., 2014), caries (Kanasi et al., 2010), and endodontic infections (Siqueira and Rôças, 2006). It is a small Gram-positive, non-motile, non-spore-forming, oxidase-, and catalase-negative, facultatively anaerobic coccus. As previously members of Nutritionally Variant Streptococci and later of genus *Abiotrophia*, the current taxonomy separates three *Abiotrophia* species under a novel genus, *Granulicatella* (Collins and Lawson, 2000). The nutritional requirements of *G. adiacens* are complex and pyridoxal or L-cysteine in the growth medium is essential for normal growth. Absence of these supplements results in elongated cell morphology (Karched et al., 2015) and an altered protein expression (Karched et al. unpublished data).

Protein secretion helps bacteria in their normal growth and physiology, e.g., nutrient acquisition, but it can also function as a virulence mechanism in host colonization or by modulating host immune responses (Finlay and Falkow, 1997; Lee and Schneewind, 2001). Bacteria have devised dedicated secretory systems (Natale et al., 2008; Green and Mecsas, 2016) for protein secretion; Gram-positive species secrete mainly via general secretory system (sec-dependent) (Schneewind and Missiakas, 2012) or twin-arginine transport (Tat) pathway (Goosens et al., 2014) while Gram-negative bacteria use more complex secretory systems (Type I to Type VIII) (Costa et al., 2015). Little knowledge is available of the protein secretion of *Granulicatella* species. Recently, a close phylogenetic relative of *G. adiacens, Granulicatella elegans*, was shown to secrete arginine deiminase, which through citrullination inhibits proliferation of human peripheral blood mononuclear cells *in vitro* (Kanamoto et al., 2007), but also inhibits biofilm surface attachment of certain dental plaque bacteria (Abdullah et al., 2013) and may associate with the pathogenesis of periodontitis and certain systemic diseases (Olsen et al., 2018). In this study, we investigated the components of the secretome of *G. adiacens*. We also obtained preliminary information on the immunoinflammatory response induced by the secretome of *G. adiacens*.

## Methods

### Bacteria and Culture Conditions

Reference bacterial strain *G. adiacens* CCUG 27809 was cultured on chocolate blood agar (CBA) with 0.001% pyridoxal hydrochloride for 2 days at 37°C and in 5% CO_2_ in air as we previously reported (Christensen and Facklam, 2001; Karched et al., 2015).

### Extracellular Protein Release

A loopful (1 μl) of bacterial colonies harvested from CBA plates were inoculated into 5 ml brucella broth (supplemented with 0.001% pyridoxal hydrochloride) and incubated in 5% CO_2_ in air at 37°C. No-bacteria control was incubated in parallel. After 24 h (in exponential growth phase) broth cultures were centrifuged at 5,000 × g for 5 min. Viability of bacteria was checked by culturing a 100-μl aliquot on CBA. Supernatants containing extracellularly released proteins were separated and filtered through 0.2 μm sterile filter to remove residual bacterial cells. All experiments were performed in duplicates and were repeated three times.

### Preparation of Secretome

The replicates of extracellular proteins released in supernatant broth samples were extracted by tri-chloroacetic acid (TCA) precipitation method as described previously (Deatherage Kaiser et al., 2015) with modifications. One volume of TCA stock (100% w/v) was mixed with four volumes of supernatant culture broth and incubated for 30 min at −20°C. Extracted proteins in broth were recovered in pellet form by centrifugation at 14,000 × g for 20 min at 4°C on Beckman J2-M1 High-Speed centrifuge. The pellet was washed twice with 0.5 ml cold acetone to remove traces of acid followed by complete air-drying in a fume hood. Desalting of the samples was achieved by diluting protein samples to 0.5 ml volume (each time) in lysis buffer and washing three times by ultrafiltration through 3K Ultra-0.5 centrifugal filter devices (Amicon) at 14,000 × g for 15 min at 4°C. Flow through were discarded and concentrates in the columns were finally eluted from columns in upside down position on collection tubes by centrifugation at 1,000 × g for 2 min at 4°C. A “no-bacteria” broth control which was incubated in parallel was used as negative control.

### Bacterial Cell Lysate/Whole Cell Protein Preparation

Harvested colonies from CBA plates were washed once in sterile PBS, followed by centrifugation at 5,000 × g for 5 min. The pellet recovered was resuspended in lysis buffer containing 1 mg/ml lysozyme and 1 mM phenyl methyl sulfonyl fluoride (PMSF) and incubated for 4 h at 4–8°C. The samples were then sonicated in Omni Ruptor at a pulse rate 40 for 8 times (1 min sonication with 1 min interval on ice). Cell lysates after sonication were centrifuged at 10,000 × g for 10 min at 4°C. Whole cell lysate of *G. adiacens* was prepared to use as a control in western blot analysis along with extracellular protein extract of the same.

### Determination of Protein Concentration

Protein concentrations in extracellular protein extract and cell lysate were estimated by Quick Start^TM^ Bradford protein microplate standard assay (Bio-Rad) as per manufacturer instructions.

### SDS-PAGE

For SDS-PAGE analysis, protein samples were mixed with 5× Laemmli sample buffer (125 mM tris, pH 6.8; 6% glycerol, 2% SDS; 5% beta-mercapthoethanol; 0.025% bromophenol blue) followed by boiling at 95°C for 5 min. After cooling at room temperature, samples were loaded on a 15% SDS-PAGE gel [4% stacking gel (4% acrylamide; 68 mM tris, pH 6.8; 0.2% SDS), 15% separating gel; 375 mM tris, pH 8.8; 0.1% SDS]. Electrophoresis was run at 150 V for 75 min (Mini-protein II Dual Slab Cell, Bio Rad). After the run, protein bands were visualized using coommassie blue.

### Western Blot Analysis

To rule out the possibility of cell lysis of *G. adiacens* cells and the release of cellular proteins in secretome preparations, western blot analysis of whole cell lysate and secretome preparation was performed. Proteins were transferred from the gel onto a PVDF membrane using Trans-Blot® Turbo™ transfer system (Bio-Rad). Membrane was blocked with 5% skimmed milk overnight at 4°C. An antibody against the cytoplasmic marker protein, Ftsz (Filamenting temperature sensitive mutant z) (Agrisera AB, Sweden) was used as a primary antibody at 1:1,000 dilution and incubated on a shaker for 1 h at room temperature. The membrane was then incubated as above with a peroxidase conjugated goat antirabbit IgG F (ab') 2 s Ab secondary antibody (1:5,000). The membrane was washed between each antibody treatment with tris-buffer saline containing Tween-20 (TBST). The membrane was finally treated with SuperSignal^TM^ West Pico chemiluminescence substrate (Pierce) and images were acquired in G:Box Imaging System (Syngene).

### nanoLC-ESI-MS/MS

Protein identification using nanoLC-ESI-MS/MS was performed by Proteome Factory (Proteome Factory AG, Berlin, Germany). The LC-MS system consisted of an Agilent 1100 nanoHPLC system (Agilent, Waldbronn, Germany), PicoTip electrospray emitter (New Objective, Woburn, MA) and an LTQ-FT Ultra mass spectrometer (ThermoFisher Scientific, Bremen, Germany). Replicate samples from secretome preparations were pooled and 400 ng protein were reduced, alkylated and digested by trypsin (Promega, Mannheim, Germany) and applied to nanoLC-ESI-MS/MS. Peptides were trapped and desalted on the enrichment column (Zorbax SB C18, 0.3 × 5 mm, Agilent) for 5 min using 1% acetonitrile/0.5% formic acid as eluent, then peptides were separated on a Zorbax 300 SB C18, 75 μm × 150 mm column (Agilent) using an acetonitrile/0.1% formic acid gradient from 5 to 40% acetonitrile within 120 min. MS spectra were automatically recorded by the mass spectrometer according to manufacturer's instrument settings for nanoLC-ESI-MSMS analyses. Proteins were identified by submitting all MS/MS spectra to the Mascot search engine (Matrix Science, London, England) and non-redundant protein database; NCBI-nr (National Center for Biotechnology Information, Bethesda, USA, version 20151202) and taxonomy Bacteria including 54,860,673 sequences. Ion charge in search parameters for ions from ESI-MS/MS data acquisition were set to “1+, 2+, or 3+.” Search parameters were as following: Fixed modifications: Carbamidomethyl (C); variable modifications: Deamidated (NQ), Oxidation (M); Peptide Mass Tolerance: ± 5 ppm; Fragment Mass Tolerance: ± 0.6 Da; Missed Cleavages: 2. Only peptides matched with a score of 20 or above were accepted and included in protein identification.

### Bioinformatics Analyses of the Secreted Proteins

The signal peptides in the secreted proteins were determined by using SignalP (http://www.cbs.dtu.dk/services/SignalP/) (Bendtsen et al., 2004) Phobius (http://phobius.sbc.su.se/) (Kall et al., 2007), and PSORTb (http://www.psort.org/psortb/) (Yu et al., 2010) and a most-votes approach was used to interpret the results. To identify lipoproteins, LipoP (http://www.cbs.dtu.dk/services/LipoP/) and PRED-LIPO (http://bioinformatics.biol.uoa.gr/PRED-LIPO/input.jsp) (Bagos et al., 2008) were used to search for lipoboxes. TatP (http://www.cbs.dtu.dk/services/TatP/) (Bendtsen et al., 2005b) and TatFind (http://signalfind.org/tatfind.html) (Rose et al., 2002) were used to predict proteins secreted via Twin-arginine translocation (Tat) pathway. To identify proteins secreted by non-classical secretory system, SecretomeP2.0 was used (http://www.cbs.dtu.dk/services/SecretomeP/) (Bendtsen et al., 2005a). Proteins that were also positive for signal peptide were disregarded. Transmembrane alpha helices were predicted combining the tools TMHMM v2.0 (http://www.cbs.dtu.dk/services/TMHMM/) (Krogh et al., 2001), SVMtm (http://ccb.imb.uq.edu.au/svmtm/) (Yuan et al., 2004), and SOSUI (http://harrier.nagahama-i-bio.ac.jp/sosui/sosuiG/sosuigsubmit.html) (Hirokawa et al., 1998). A theoretical 2-dimensional electrophoresis (2-DE) image of the secreted proteins was acquired using the software JVirGel vs. 2.0 (Hiller et al., 2003).

For functional annotation of the secreted proteins, Gene Ontology (GO) IDs and terms were assigned using the tool “WEGO” (http://wego.genomics.org.cn/) (Ye et al., 2018) and the program Blast2GO. Proteins with enzymatic functions, possibly involved in metabolic pathways, were predicted by KEGG Pathway Database (http://www.genome.jp/kegg/pathway.html). Putative virulence proteins were predicted by the software “VirulentPred” (http://203.92.44.117/virulent/) (Garg and Gupta, 2008), and by using Virulence Factor DataBase (VFDB, http://www.mgc.ac.cn/VFs/) (Chen et al., 2016). The secreted proteins were grouped into functional protein association networks using STRING (https://string-db.org/) (von Mering et al., 2005). MultitaskProtDB (http://wallace.uab.es/multitask) was used for identifying “moonlighting proteins” in the *G. adiacens* secretome (Hernández et al., 2014).

### Isolation of Human Peripheral Blood Mononuclear Cells (PBMCs)

Ethical approval for blood collection from a healthy human volunteer was obtained from Health Science Center Ethical Committee, Kuwait University. Peripheral blood mononuclear cells (PBMCs) were isolated from the blood of a systemically healthy human volunteer as described earlier (Fuss et al., 2009; Bhardwaj et al., 2018). Blood was collected by venipuncture into tubes containing heparin vacutainer (4 ml /tube). PBMCs were fractionated by Ficoll-Paque density gradient centrifugation method. Under careful aseptic conditions, the blood was carefully layered over the Ficoll-Paque^TM^Plus (GE Healthcare) solution in the test tube. The tube was centrifuged at 3,400 rpm at room temperature for 10 min and the resulting buffy coat layer containing PBMCs was transferred to another clean tube. After washing twice in 5 ml of RPMI medium the tube was centrifuged at 2,000 rpm for 5 min to recover the cell pellet. The supernatant was discarded and the cell pellet was finally resuspended in 1 ml of RPMI medium (supplemented with 10% heat-inactivated fetal bovine serum and 2% of Gibco^TM^ 100× antibiotic-antimycotic solution). Cells were enumerated using 10 μl of homogenous cell suspension in hemocytometer under ×400 magnification of the microscope.

### PBMCs Treatment With Secretome

PBMCs were stimulated with secretome preparations for 24 h. One hundred microliter of the secretome preparation was added into each well-containing 0.5 ml of PBMCs (10^6^ cells per ml). The plate was incubated for 24 h in 5% CO_2_ in air at 37°C. PBS, which was the medium for secretome, was used as a negative control.

### Cytokine Profiling Using the Membrane Arrays

Cytokines produced by PBMCs on stimulation with *G. adiacens* secretome were detected using a human cytokine array kit (Proteome Profiler^TM^ Antibody Arrays R&D Systems^TM^). Nitrocellulose membrane with 36 selected capture antibodies spotted in duplicate was used to determine the relative levels of cytokines. The array membrane was blocked with assay buffer for 1 h at room temperature to prevent non-specific binding. The secretome-stimulated PBMC sample (1.5 ml) was diluted in assay buffer with 15 μl of reconstituted human cytokine array detection antibody cocktail and incubated at room temperature for 1 h. Following three washes in wash buffer, the array was treated with streptavidin HRP for 30 min at room temperature on a rocking platform shaker. Washed array was finally incubated with chemiluminescence reagent for 10 min and images were acquired in Syngene G:Box Imaging System. The positive signals seen on the array were identified by comparing it with the transparency overlay template with the pairs of reference spots in three corners of each array. Pixel densities (signals) in each spot on the array were collected, mean spot pixel density was created and analyzed image analysis software provided with G:Box Imaging System. The experiments were run in duplicates and repeated three times.

## Results

### Analysis of the Secretome of *G. adiacens*

**Figure 1:**
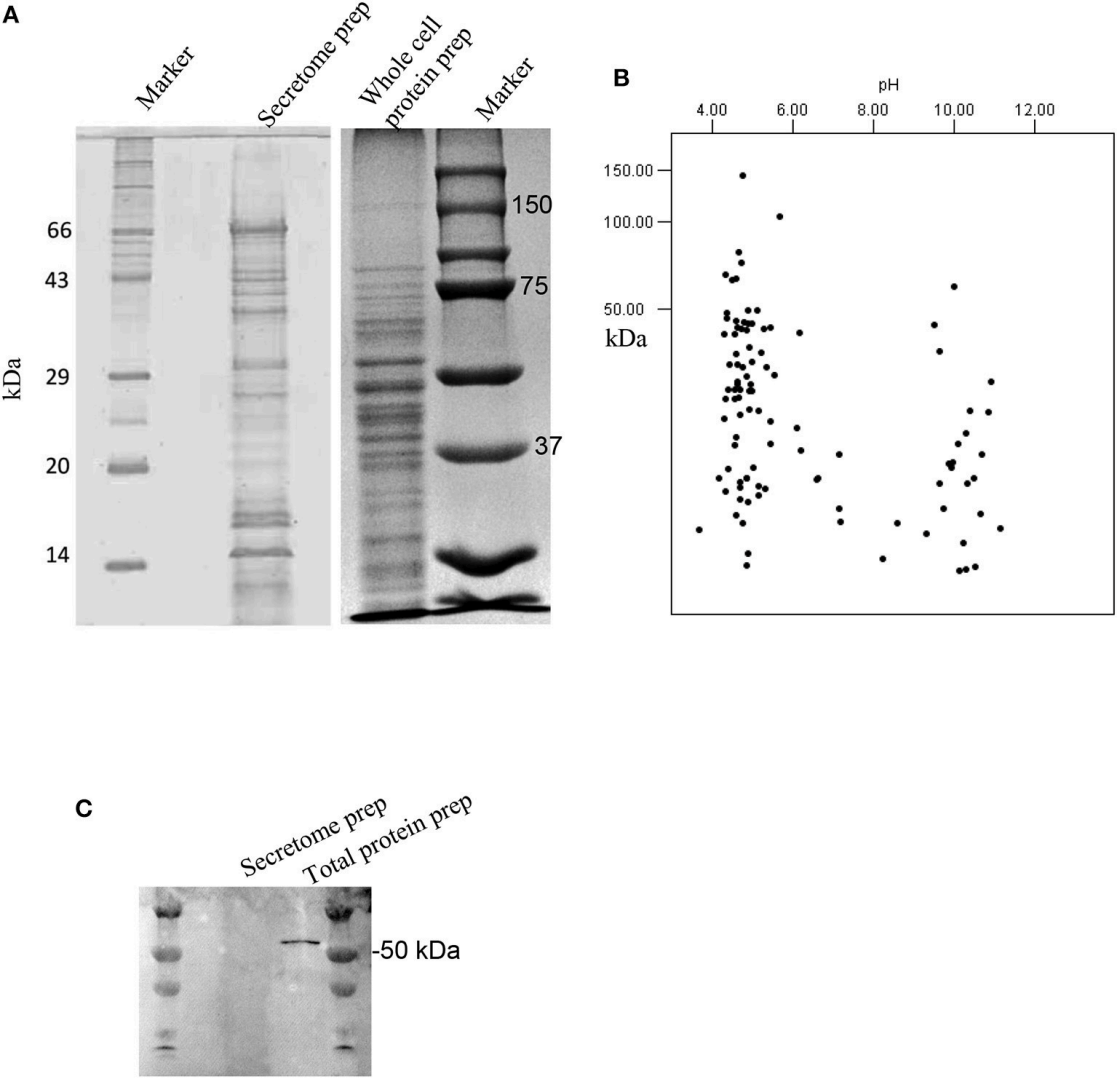
Analysis of the secretome of *G. adiacens*. **(A)** SDS-PAGE gel showing protein bands from secretome preparation. **(B)** Protein sequences from LC-MS analysis of the secretome were analyzed by an *in silico* 2DE tool. **(C)** Western blot analysis showing the absence of the cytoplasmic marker protein FtsZ in the secretome preparation.

Secretome preparation (Figure 1A) from *G. adiacens* was analyzed by LC-MS/MS. Database search (NCBI-nr) revealed 101 proteins (Table S1). As depicted in a theoretical 2DE map of the secretome, the MW of the secreted proteins ranged between 3.7 and 148 kDa (Figure 1B). The secretome proteins formed two clusters with respect to predicted isoelectric point (pI) values. Majority of the proteins belonged to the cluster with the pI range of 4.0–5.5, while the other cluster was of the proteins between pI values 9.5 and 11. To exclude the possibility that the secretome preparation contained proteins that originated from bacterial cell lysis, we used in western blot analysis a cytoplasmic cell lysis marker, Fts-Z protein, which was detected only in the total protein preparation from *G. adiacens* but not in the secretome preparation (Figure 1C). Further, plating of an aliquot of the 24-h broth culture confirmed the viability of bacteria during the experiment. To determine subcellular localization of the 101 secreted proteins detected with LC-MS/MS, PsortB tool was used. We found that 63 proteins were predicted to be cytoplasmic (60%), 10 cytoplasmic membrane (9.6%), 6 extracellular (5.7%), 2 cell wall anchored (1.9%), whereas the localization of 23 proteins (22%) could not be predicted.

Protein sequences of the secretome of *G. adiacens* were analyzed for the route of their extracellular release by various bioinformatics tools as described in the methods section. As predicted by SignalP tool, 18 of the secreted proteins were predicted to possess signal sequence, suggesting the “Sec” pathway for their secretion. TatP prediction tool showed that 8 of the sequences contained TatP signal sequence. There were a total of 9 lipoproteins (Pred-Lipo, LipoP) in the secretome. Using the tool SecretomeP 2.0, 31 sequences were predicted to be secreted via non-classical secretion pathway. However, since 15 of them contained a signal sequence and hence concluded to be secreted via Sec pathway, those were discarded and only 16 proteins were finally considered to be secreted via non-classical pathway. Transmembrane alpha helices were found in 9 proteins. Two of the 9 protein sequences that were predicted to have at least 2 transmembrane domains were considered as putatively membrane attached and therefore not included in further analyses. Table 1 lists the 16 proteins from the secretome which were identified as “moonlighting proteins.”

**Table 1:** List of *G. adiacens* secretome proteins with a predicted moonlighting function.

**GI number**	**Protein**
gi|491802570	Serine protease
gi|491797953	Molecular chaperone DnaK
gi|491800441	Superoxide dismutase
gi|491800797	Glyceraldehyde-3-phosphate dehydrogenase
gi|491800365	NADH oxidase
gi|748591028	30S ribosomal protein S20
gi|491799730	Short-chain dehydrogenase
gi|491801600	50S ribosomal protein L7/L12
gi|491802592	30S ribosomal protein S6
gi|259036192	Thioredoxin
gi|50902517	SSU ribosomal protein S19P
gi|491801111	50S ribosomal protein L30
gi|491801148	Elongation factor Tu
gi|491801605	50S ribosomal protein L10
gi|491799115	50S ribosomal protein L32
gi|259035990	Phosphoglycerate kinase

### Potential Virulence Proteins in *G. adiacens* Secretome

Virulence potential of the *G. adiacens* secretome was assessed by manually searching for their associations with virulence activities in other species, since little is known of the virulence factors of *G. adiacens*. Additionally, *in silico* prediction of virulence factors was performed using the online tools “VirulentPred” and “VFDB” (Virulence Factor DataBase). Table 2 shows the list of 22 proteins from the secretome that were deduced from *in silico* prediction and/or based on evidence from the literature. Thioredoxin, serine proteinase, aminopeptidase, molecular chaperones DnaK and GroES, Superoxide dismutase, N-acetylmuramoyl-L-alanine amidase, Glyceraldehyde-3-phosphate dehydrogenase, phosphoglycerate kinase, and acyl carrier protein are the major proteins with demonstrated virulence properties in other bacterial species.

**Table 2:** Putative virulence factors identified in *G. adiacens* secretome.

**GI number**	**Protein**	***In silico* prediction**	**Literature evidence**
gi|491802570	Serine protease	Yes	Yes (Ruiz-Perez and Nataro, 2014)
gi|491801087	Aminopeptidase	Yes	Yes (Carroll et al., 2012)
gi|491800441	Superoxide dismutase	Yes	Yes (Gerlach et al., 1998)
gi|491797953	Molecular chaperone DnaK	Yes	Yes (Goulhen et al., 1998)
gi|259036192	Thioredoxin	Yes	Yes (Bjur et al., 2006)
gi|491800365	NADH oxidase	No	Yes (Ge et al., 2016)
gi|491798572	N-acetylmuramoyl-L-alanine amidase	Yes	Yes (Romero et al., 2004)
gi|491800929	Molecular chaperone GroES	Yes	Yes (Hinode et al., 1995)
gi|491800797	Glyceraldehyde-3-phosphate dehydrogenase	Yes	Yes (Lu et al., 2009)
gi|491797310	Acyl carrier protein	Yes	Yes (Feng et al., 2015)
gi| 259036239	Phosphocarrier protein	No	Yes (Dubreuil et al., 1996)
gi|259035990	Phosphoglycerate Kinase	No	Yes
gi|491799853	DNA starvation/stationary phase protection protein	Yes	Yes (Loprasert et al., 2004)
gi|491800219	CHAP domain-containing protein	Yes	Yes (Zhong et al., 2014)
gi|491801017	LysM peptidoglycan-binding domain-containing protein	Yes	Yes (Shi et al., 2016)
gi|491796985	YlbF family regulator	Yes	Yes (Tortosa et al., 2000)
gi|491798894	CsbD family protein	Yes	Yes (Lanotte et al., 2013)
WP_049555432	PTS ascorbate transporter subunit II	Yes	Yes (Afzal et al., 2015)
gi|259035249	WXG100 family type VII secretion target	Yes	Yes (Pallen, 2002)
gi|491797708	Cysteine desulfurase	Yes	Yes (Großhennig et al., 2016)
gi|491798949	Agglutinin receptor	Yes	No
gi|259035137	YbaB/EbfC family protein	Yes	Yes (Jutras et al., 2012)

### Gene Ontology Analysis

**Figure 2:**
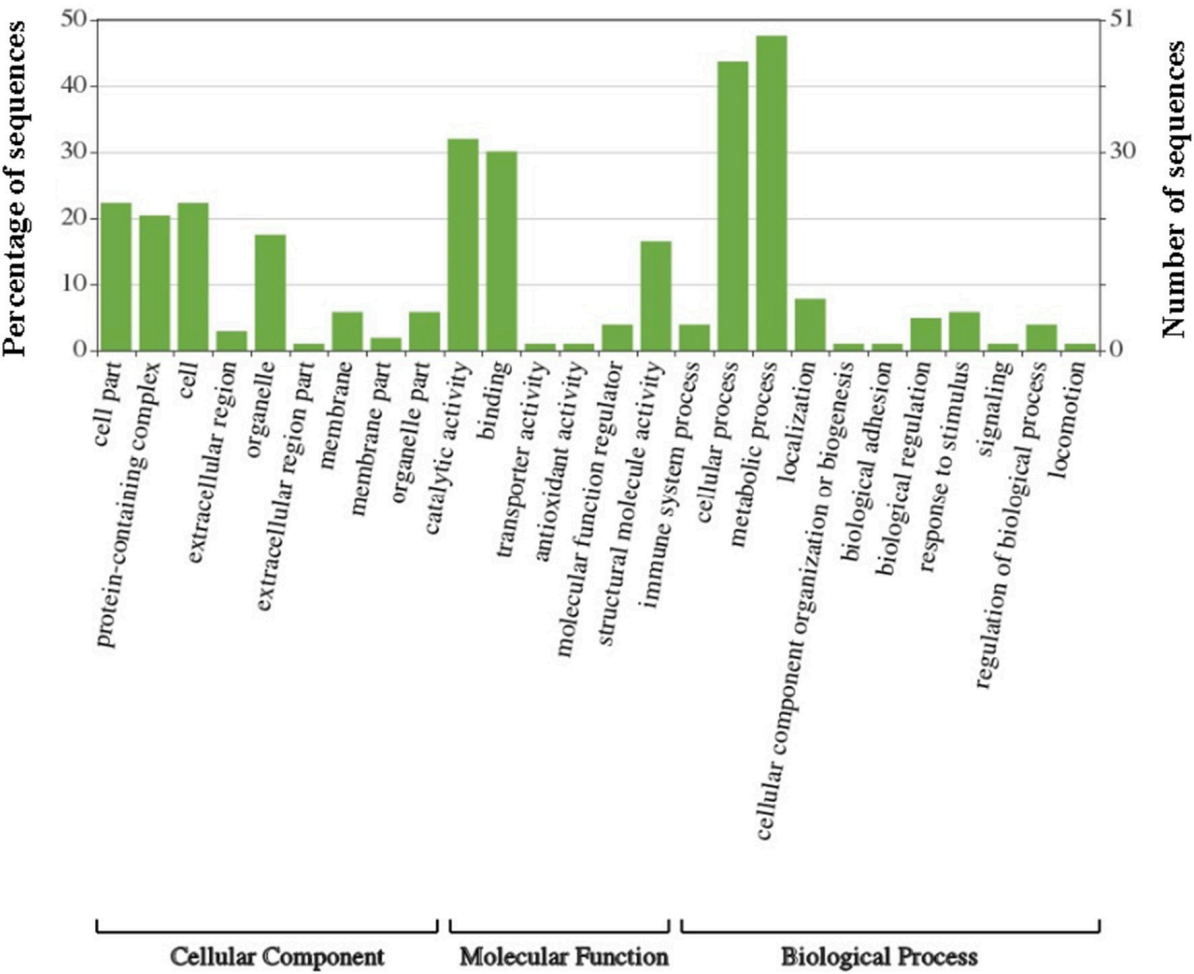
Gene Ontology analysis of *G. adiacens* secreted proteins. Gene ontology annotation was achieved using Blast2GO and an online software “WEGO.” Protein sequences were grouped into 3 categories based on their properties and functions.

Gene Ontology (GO) analysis of the amino acid FASTA sequences of the *G. adiacens* secretome was achieved by using the tools Blast2GO and WEGO. In the case of WEGO, the XML file from InterPro analysis was used (Figure 2). Overall, 73 of the 104 sequences were assigned with GO annotation. The secreted proteins were divided into 3 groups based on GO terms: 63 proteins in “biological process,” 31 proteins in the “cellular component” group, and 65 proteins in the group “molecular function.”

### KEGG Pathway Analysis

**Figure 3:**
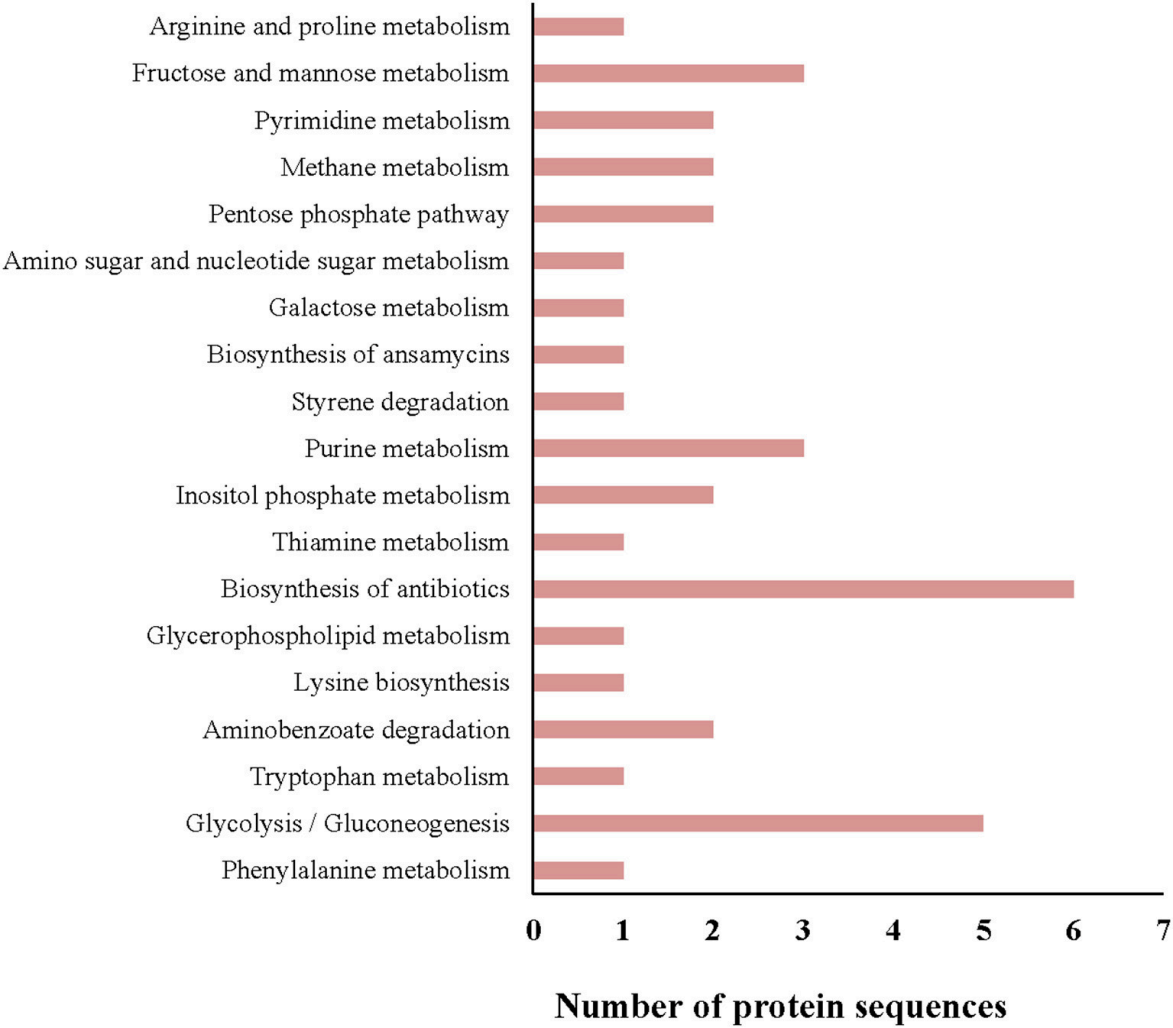
KEGG pathway analysis of the *G. adiacens* secretome. All protein sequences from the secretome were analyzed by KEGG pathway tool, which was included as a “plugin” tool within Blast2GO software.

All protein sequences from the *G. adiacens* secretome were subjected to KEGG pathway annotation and analysis (Figure 3). Of all the pathways identified, 6 proteins were predicted to be involved in the biosynthesis of antibiotics, followed by 5 proteins that occurred in the glycolysis pathway. Fructose and mannose metabolism, and purine metabolism pathways contained 3 proteins each. One of the secreted proteins, transketolase, was predicted to be occurring in the biosynthesis of ansamycins, a family of bacterial secondary metabolites with antimicrobial activity.

### Functional Protein Association Network Analysis

**Figure 4:**
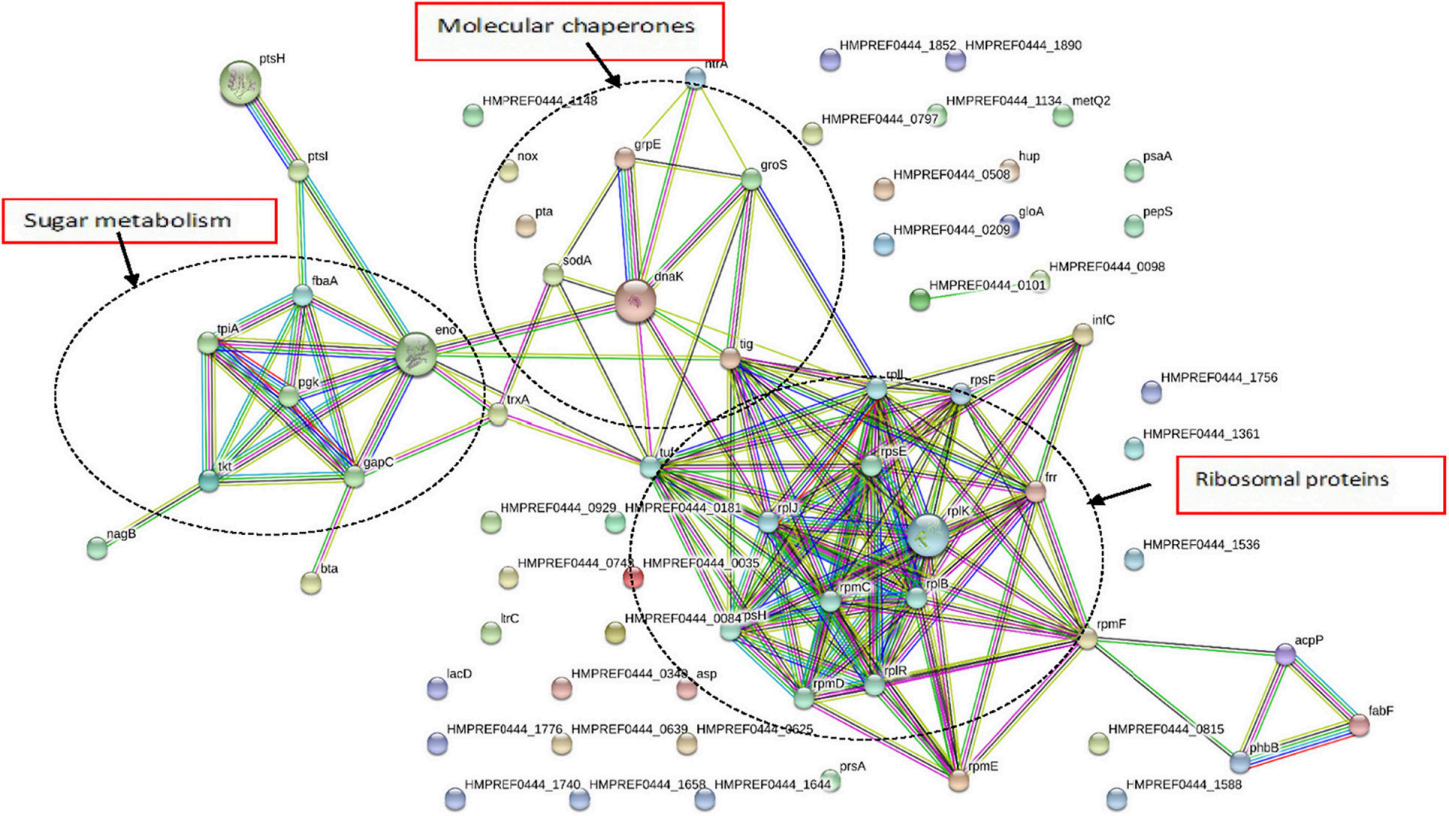
Functional protein association networks of *G. adiacens* secretome. The online tool STRING was used for grouping the secreted proteins on the basis of functional networks. Minimum interaction scores were set at a strong confidence level of 0.7. The three major network groups formed are shown in dotted circles. Seven different colored link a number of nodes and represent seven types of evidence used in predicting associations. A red line indicates the presence of fusion evidence; a green line represents neighborhood evidence, a blue line represents co-occurrence evidence; a purple line represents experimental evidence; a yellow line represents textmining evidence; a light blue line represents database evidence and a black line represents coexpression evidence.

As seen in Figure 4, *G. adiacens* secretome proteins formed three major groups in the STRING network, i.e., sugar metabolism, ribosomal proteins and heat shock proteins/chaperones. Components of the sugar metabolism network were phosphoglycerokinase, enolase, triose phosphate isomerase, Fructose-1,6-bisphosphate aldolase, and phosphocarrier protein. Putative virulence-associated proteins super oxide dismutase, thioredoxin, molecular chaperones (DnaK, GroS, and GrpE) NADH oxidase and HtrA, a trypsin-like protease, formed another cluster. The ribosomal protein group consisted mainly of the secreted ribosomal proteins (Figure 4).

### Cytokine Stimulation by *G. adiacens* Secretome Preparation

**Figure 5:**
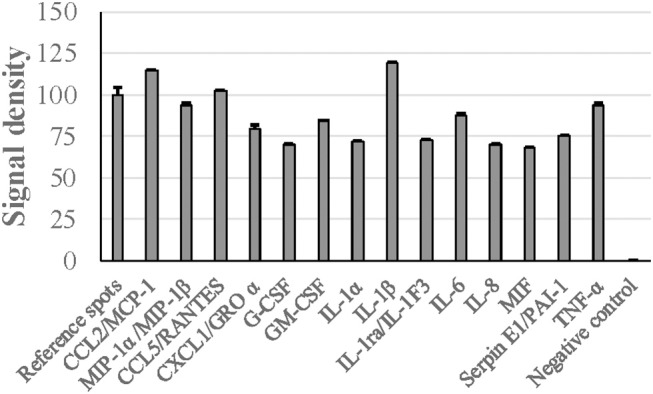
Cytokine induction from human PBMCs by *G. adiacens* secretome preparation. Fractionated human PBMCs were stimulated by *G. adiacens* secretome preparation for 24 h. The cytokines produced were detected by using Proteome Profiler™ membrane array. Means (SD) of signal densities of spots were determined using Gene Tools analysis software in Syngene Imaging System.

When human PBMCs were stimulated with *G. adiacens* secretome preparation, semiquantitative analysis showed that IL-1β and MCP-1 were the cytokines found at highest relative quantities, followed by TNF-α and RANTES (Figure 5). Other important cytokines detected were IL-8, IL-6, G-CSF, GM-CSF, MIP1-α, and MIP1-β. When the PBMCs were stimulated with total proteins of *G. adiacens*, CCL-1, CCL2 (MCP-1), and G-CSF were not detected (data not shown).

## Discussion

While protein secretion is a well-established virulence mechanism in bacteria, little is known of the secretome of *G. adiacens*. Recently, *G. elegans*, a close phylogenetic relative of *G. adiacens*, was shown to secrete arginine deaminase (Kanamoto et al., 2007), a citrullinating enzyme that was shown to inhibit proliferation of human PBMCs *in vitro* and may associate with pathogenesis of periodontal and certain systemic diseases (Olsen et al., 2018). In this study, we took a qualitative proteomics approach to obtain a protein profile of the *G. adiacens* secretome. Since the objective of this study was to identify the proteins in the *G. adiacens* secretome by mass spectrometry, qualitative proteomics was adequate (Zijnge et al., 2012; Bao et al., 2015, 2017; Mohammed et al., 2017; Suriyanarayanan et al., 2018) without the necessity for validating the identified proteins as is the case for quantitative proteomics. We found that the secretome was enriched with a large number of putative virulence factors utilizing various bioinformatics analysis tools, we were able to characterize most of the secretome proteins *in silico*. The secretome proteins were predicted to be released via various secretion systems such as, sec-dependent, Tat pathway and via a non-classical secretory system.

To rule out the possibility of contamination of the secretome with subcellular proteins we used an established cytoplasmic marker protein Fts-Z (Terrasse et al., 2015) which remained absent in all *G. adiacens* secretome preparations.

By combining *in silico* analysis with experimental evidence and available bibliography, we were able to identify more than 20 putative virulence-associated proteins in *G. adiacens* secretome. This is in line with secretomes of other much-studied oral bacteria such as *A. actinomycetemcomitans* (Zijnge et al., 2012) and *Porphyromonas gingivalis* (Stobernack et al., 2016) Remarkably, several well-known virulence factors in other bacteria, such as serine protease, thioredoxin, superoxide dismutase, phosphocarrier and acyl carrier proteins were also detected in the *G. adiacens* secretome. Superoxide dismutase converts superoxide anions into oxygen and hydrogen peroxide. In streptococci, superoxide dismutase is displayed on the cell surface as well as released extracellularly (Gerlach et al., 1998; McMillan et al., 2004). Since *G. adiacens* is a catalase-negative organism, SOD might play an important role in the detoxification of oxidative burst against them by the host cells. *G. adiacens* apparently is equipped with more strategies to survive during oxidative stress. Indeed, gpoA gene, encoding for glutathione peroxidase was found in *G. adiacens* genome (Sequence ID WP_005604890.1). Superoxide dismutase is required not only for H_2_O_2_ resistance in *S. mutans*, but also is needed for coexistence with *S. sanguinis* (Fujishima et al., 2013). Whether SOD plays such a role in *G. adiacens* needs to be studied.

Virulence potential of several other proteins in *G. adiacens* secretome has been studied previously. For example, *S. mutans, S. sanguinis* and other species require NADH oxidase for biofilm formation (Ge et al., 2016). Streptococci secrete glyceraldehyde-3-phosphate dehydrogenase (GAPDH), which is known to help during bacterial invasion (Nelson et al., 2001). Serine proteases cause cytopathic effects and exhibit enterotoxin activity. They degrade mucins, including leukocyte surface O-glycoproteins with vital roles in numerous cellular functions, resulting in advantage for mucosal colonization and immune modulation (Dutta et al., 2002; Ruiz-Perez et al., 2011; Ruiz-Perez and Nataro, 2014). In *Salmonella enterica*, thioredoxin helps the bacterium in intracellular replication and virulence in a mouse model (Bjur et al., 2006). Serine protease of *Fusobacterium nucleatum* and *S. mutans* are shown to be critical in the survival and pathogenicity of these species (Diaz-Torres and Russell, 2001; Doron et al., 2014). Thus, it would be of great interest to study how *G. adiacens* utilizes this arsenal of putative virulence proteins for its own survival and to cause an infection.

Although *G. adiacens* secretome comprised of many proteins of cytoplasmic origin according to the prediction tools, interestingly, several of these belong to a so-called group “moonlighting proteins” (Jeffery, 1999; Henderson and Martin, 2014), which have a known function inside the bacterial cell but also participate in different biological processes in the extracellular medium after their secretion. That *G. adiacens* secretome consisted of several moonlighting proteins is of great significance since they are shown to play a role in bacterial virulence (Henderson and Martin, 2011; Wang et al., 2014). Major ribosomal proteins detected in *G. adiacens* secretome were 50S proteins L10, L11, L7/L12, L15, L32, and 30S proteins S5, S6, S8, and S20. Importantly, in other bacteria ribosomal protein L7/L12 is highly antigenic and immunogenic (Oliveira and Splitter, 1996; Ribeiro et al., 2002). Of the glycolytic enzymes, phosphoglycerate kinase, triose-phosphate isomerase, aldolase, and enolase possess moonlighting properties, e.g., they function as adhesins (Tunio et al., 2010), receptors for transferrin (Modun et al., 2000), neutrophil evasion proteins (Terao et al., 2006), immunomodulators (Madureira et al., 2007) and participate in extracellular polysaccharide synthesis (Lu et al., 2009) Oral bacteria express a number of molecular chaperones, including DnaK (hsp60) and GroES (GroEL) found in *G. adiacens* secretome. They express on the cell surface to use them as adhesins and can release them into the extracellular milieu to act as signaling virulence factors (Hinode et al., 1995; Goulhen et al., 1998; Henderson et al., 2006). The multifunctioning potential of moonlighting proteins may help *G. adiacens* propagate in its natural habitats as well as in sterile body areas.

More than 60 of *G. adiacens* secretome proteins were grouped into biological processes and molecular functions ontology groups. When we obtained GO annotations for the *G. adiacens* whole genome, we found that about 1,000 predicted proteins were grouped into “molecular functions,” followed by about 900 and 400 proteins in the groups “biological processes” and “cellular composition,” respectively (data not shown). The secretome proteins mapped to 19 different KEGG pathways, with antibiotic biosynthesis and glycolysis being most represented. The antibiotic biosynthesis pathway consisted of the enzymes phosphopyruvate hydratase, transketolase, glycolaldehyde transferase, triosephosphate isomerase, aldolase, and phosphoglycerate kinase, several of which have been experimentally shown to be essential for antibiotic biosynthesis (Barnard-Britson et al., 2012; Liu et al., 2016). Other prominent pathways were purine metabolism, fructose metabolism and aminobenzene degradation. These results suggest that *G. adiacens* secretome proteins with metabolic activities might help the bacterium in utilizing nutrients available in the extracellular milieu (Cezairliyan and Ausubel, 2017).

Functional associations among the secreted proteins using STRING network analysis showed three distinct network groups, i.e., sugar metabolism, ribosomal proteins, and putative virulence factors. Enzymes involved in sugar metabolism, i.e., phosphoglycerate kinase, triose-phosphate isomerase, 2-phosphoglycerate dehydratase, transketolase formed a cluster. Several of these enzymes seem to have overlapping functions, i.e., they were also involved in antibiotic biosynthesis as predicted by KEGG. In the cluster that consisted of putative virulence factors, molecular chaperone DnaK showed interactions with other chaperones GrpE, GroS, thioredoxin, and PPlase. This group also showed interactions with other virulence factors such as superoxide dismutase and trypsin-like protein HtrA. Molecular chaperones aid bacterial pathogenesis by helping bacteria in coping with stressful host environment, e.g., acidified phagosome, oxidative burst, and phagosome fusion with lysosomes (Hosogi and Duncan, 2005). Further, chaperones are potent immunogens and possess direct activating effect on different cell populations including lymphoid, myeloid, vascular endothelial and bone cells (Lewthwaite et al., 1998).

In several pathogenic bacteria, secreted proteins are known to modulate host immune responses (Finlay and Falkow, 1997; Lee and Schneewind, 2001). To get preliminary knowledge of the cytokine stimulatory potential of the *G. adiacens* secretome, we used human PBMCs as target host cells. Major cytokines such as IL-1β, TNF-α, MCP-1 were found at high levels as evidenced by higher signal densities than the reference spots. Although cytokine induction of PBMCs from total protein preparation from *G. adiacens* was similar, MCP-1 and G-CSF were absent. This was also observed in our previous study where biofilms failed to stimulate these cytokines but the biofilm supernatants did (Bhardwaj et al., 2018). While secreted components of different bacterial species have been previously shown to elicit inflammatory response from host cells (Oscarsson et al., 2008; Dapunt et al., 2016), a protein of *Brucella suis* was able to inhibit TNF-α production from macrophages when it was released extracellularly (Caron et al., 1996). Therefore, specific stimulation of certain cytokines by secreted proteins, but not the total bacterial proteins, is suggestive of possible mechanisms *G. adiacens* might use for systemic stimulation.

In conclusion, we unraveled the secretome of *G. adiacens*, an oral bacterium well-documented in infective endocarditis, but also recently shown to be involved in oral infections. Importantly, the secretome of *G. adiacens* comprised of a large number of putative virulence factors. Of particular importance is the finding that the *G. adiacens* secretome comprised of a number of “moonlighting” proteins, which in other species are shown to enhance bacterial colonization and virulence through their multifunctional roles (Pavkova et al., 2017; Graf et al., 2019). Thus, our results provide a basis for investigating the role of secreted proteins of *G. adiacens* in oral infections as well as in infective endocarditis.

## Data Availability

Mass spectrometry data has been submitted to “PRIDE Archive” (https://www.ebi.ac.uk/pride/archive/) repository with the project accession number PXD013000. The data files can be accessed with the username: reviewer53961@ebi.ac.uk and password: VmJUzTGl.

## Ethics Statement

This study was carried out in accordance with the recommendations of the Ethical Committee of Health Sciences Center (HSC), Kuwait University. Written informed consent was obtained from the human volunteer and the consent was in accordance with the Declaration of Helsinki. The protocol was approved by the Ethical Committee of the HSC, Kuwait University.

## Author Contributions

MK conceived and designed the study, bioinformatics analyses. RB and MK performed the laboratory experiments. MK, RB, AT, and SA wrote the manuscript.

### Conflict of Interest Statement

The authors declare that the research was conducted in the absence of any commercial or financial relationships that could be construed as a potential conflict of interest.

## Supplementary Material

Table S1 List of proteins identified in *G. adiacens* secretome. Secretome preparation from *G. adiacens* was analyzed by LC-MS/MS and further revealed by database search (NCBI-nr).
